# The nucleus accumbens in reward and aversion processing: insights and implications

**DOI:** 10.3389/fnbeh.2024.1420028

**Published:** 2024-08-09

**Authors:** Ying Xu, Yingjie Lin, Ming Yu, Kuikui Zhou

**Affiliations:** School of Health and Life Sciences, University of Health and Rehabilitation Sciences, Qingdao, China

**Keywords:** addiction, aversion, depression, nucleus accumbens, reward

## Abstract

The nucleus accumbens (NAc), a central component of the brain’s reward circuitry, has been implicated in a wide range of behaviors and emotional states. Emerging evidence, primarily drawing from recent rodent studies, suggests that the function of the NAc in reward and aversion processing is multifaceted. Prolonged stress or drug use induces maladaptive neuronal function in the NAc circuitry, which results in pathological conditions. This review aims to provide comprehensive and up-to-date insights on the role of the NAc in motivated behavior regulation and highlights areas that demand further in-depth analysis. It synthesizes the latest findings on how distinct NAc neuronal populations and pathways contribute to the processing of opposite valences. The review examines how a range of neuromodulators, especially monoamines, influence the NAc’s control over various motivational states. Furthermore, it delves into the complex underlying mechanisms of psychiatric disorders such as addiction and depression and evaluates prospective interventions to restore NAc functionality.

## 1 Introduction

Reward and aversion, two major components of motivation, are essential for learning and adaptation, emotional processes, and generating goal-directed behavior. The nucleus accumbens (NAc) has been historically acknowledged for its pivotal role in reward processing and the regulation of reward-seeking behaviors (Bakshi and Kelley, 1993; Kelley et al., 1997; Soares-Cunha et al., 2020). However, more recent studies have illuminated the NAc’s substantial involvement in aversive processing as well (Levita et al., 2012; Richard et al., 2013; Soares-Cunha et al., 2020; Nishioka et al., 2023). Dysfunction of the NAc induces deficits in both approach and avoidance behaviors (Hoebel et al., 2007; Schwartz et al., 2017).

The NAc’s function in the dichotomous processing of reward and aversion is intricate and dynamic (Lobo et al., 2010; Xiu et al., 2014; Hu, 2016), and its subregions appear to have distinct roles (Al-Hasani et al., 2015; Yang et al., 2018). Neurons within these subregions exhibit further heterogeneity in both the transcription and connectivity levels (Castro and Bruchas, 2019; Chen et al., 2021). Studies employing genetic engineering and optogenetic approaches have demonstrated that different NAc pathways might exert opposing functions over motivated behaviors (Yang et al., 2018; Soares-Cunha et al., 2022). Recently, the development of genetically encoded calcium indicators and fluorescent probes has enabled real-time tracking of neuronal activity and neurotransmitter dynamics in the NAc in freely moving animals (Sun et al., 2020; Chen et al., 2023; Zhuo et al., 2024). This review describes the latest advancements in comprehending the specific role of NAc neuronal ensembles or pathways in mediating reward and aversion and discusses the involvement of the NAc in psychiatric disorders such as addiction and depression (Russo and Nestler, 2013).

## 2 Behavioral processes modulated by the NAc

The NAc contributes to a variety of behavioral processes, including motivation, reward processing, reinforcement learning, and decision-making. Motivation, which involves the drive to pursue goals, is modulated by the NAc, influencing both the anticipation and consumption of rewarding stimuli (Berridge and Kringelbach, 2015). Reward processing, crucial for evaluating and encoding the hedonic value of outcomes, heavily relies on the NAc as a central hub for integrating information about rewarding stimuli (Luscher and Malenka, 2011). Reinforcement learning, the process of updating action–outcome associations based on experienced outcomes, is influenced by the NAc (Schultz, 2016). Moreover, decision-making, which entails selecting between available options, is modulated by the NAc through integrating information about the value, effort, and costs associated with different choices (Floresco, 2015).

## 3 NAc neurons mediate positive and negative reinforcement

The NAc is traditionally divided into two main subregions: the core and the shell (NAcC and NAcSh). NAc neurons can be classified into distinct subtypes based on their differential expression of genes, electrophysiological properties, and connectivity. Over 90% of NAc neurons are medium spiny neurons (MSNs) (Chen et al., 2021). Molecular heterogeneities in MSNs have been observed not only within the core and shell regions but also along the anterior–posterior and dorsal–ventral axes (Castro and Bruchas, 2019). Although certain genes are highly enriched in specific subregions (Voorn et al., 1989; Jongen-Relo et al., 1994), no definitive borders between these regions can be identified using marker genes (Chen et al., 2021).

**Figure 1:**
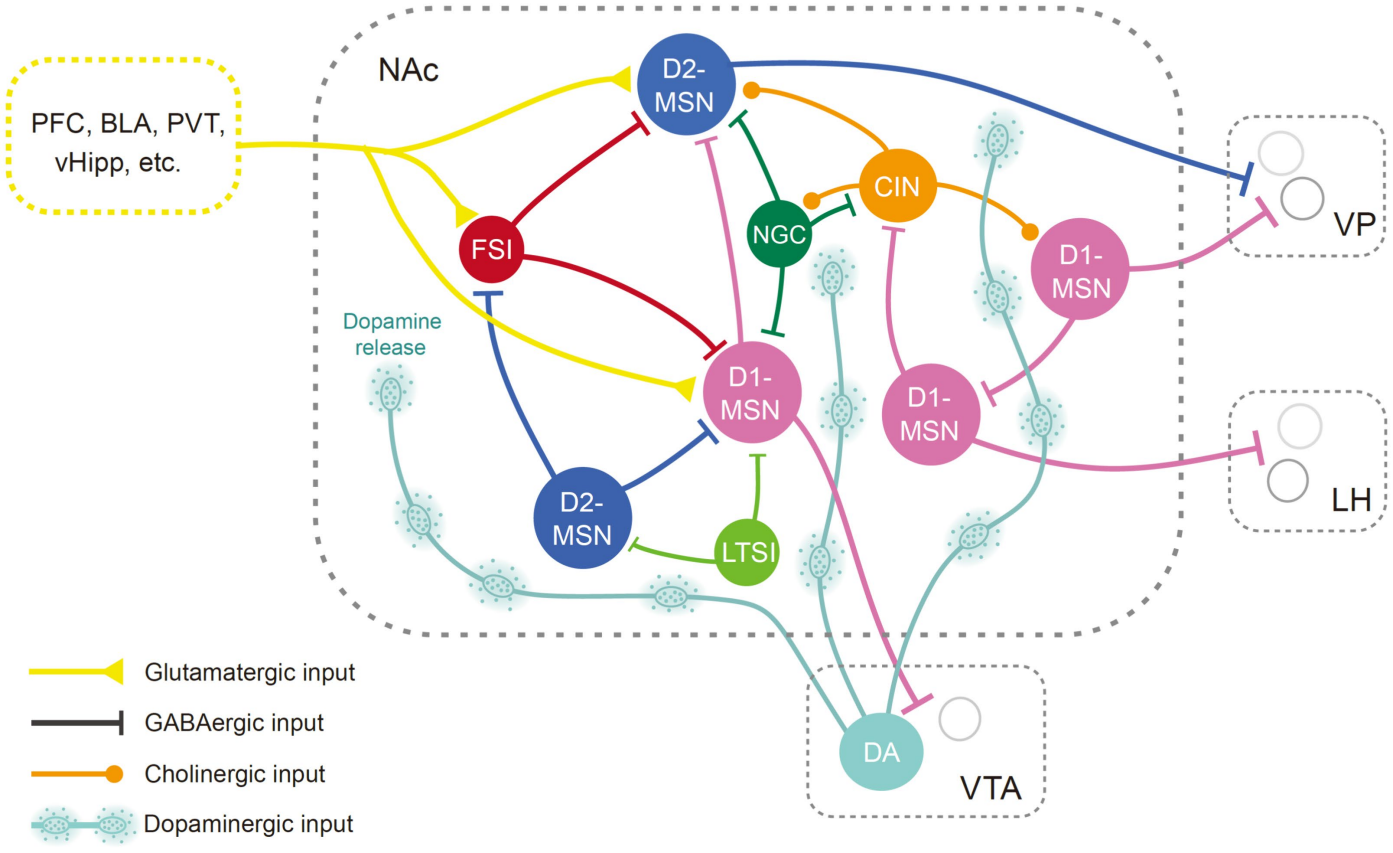
Anatomical diagram of NAc neuron types. Illustration of different NAc neuron types and their basic connectivity pattern. Neurons in the NAc receive glutamatergic inputs from the cortex, the hippocampus, the amygdala, and thalamic regions, as well as dopaminergic input from the midbrain. The MSNs, the principal neurons in the NAc, are innervated by neighboring GABAergic interneurons and cholinergic neurons. The NAc mainly targets the VP, LH, and VTA regions. BLA, basolateral amygdala; CIN, cholinergic interneuron; D1-MSN, D1 type dopamine receptor-expressing medium spiny neuron; D2-MSN, D2 type dopamine receptor-expressing medium spiny neuron; DA, dopaminergic neurons; FSI, fast-spiking interneurons; LH, lateral hypothalamus; LTSI, low-threshold-spiking interneuron; NGC, neurogliaform cells; NAc, nucleus accumbens; PFC, prefrontal cortex; PVT, paraventricular thalamus; vHipp, ventral hippocampus; VP, ventral pallidum; VTA, ventral tegmental area.

MSNs are the major projection neurons in the NAc, while interneurons might exert their function by modulating the output of the MSNs (Figure 1). Typically, MSNs are not spontaneously active and can exhibit a bistable membrane potential (O'Donnell and Grace, 1995; O'Donnell et al., 1999; Belleau and Warren, 2000; Mahon et al., 2006). Activities of MSNs are primarily triggered by glutamatergic inputs induced depolarization (O'Donnell and Grace, 1995; O'Donnell et al., 1999). Glutamatergic inputs could also enhance spike discharge via metabotropic receptors (D'Ascenzo et al., 2009). GABAergic afferents from ventral arkypallidal neurons and local accumbens interneurons restrict MSN bursting and plasticity (Owen et al., 2018; Vachez et al., 2021). Neuromodulators also significantly shape the output of MSNs (O'Donnell et al., 1999; Brady and O'Donnell, 2004).

While traditional perspectives highlight a functional dichotomy in MSNs based on differential expression of dopamine receptor-1 (D1) and dopamine receptor-2 (D2) (Shippenberg et al., 1993; Hikida et al., 2010; Lobo et al., 2010; Kravitz et al., 2012), recent research challenges this notion (Soares-Cunha et al., 2016b; Cole et al., 2018; Soares-Cunha et al., 2020). *In vivo* recordings demonstrate that MSNs respond diversely to stimuli such as natural rewards, addictive drugs, and stress (Ghitza et al., 2003; Roitman et al., 2004; Nestler, 2015; Morrison et al., 2017). The response patterns of MSNs depend on their spatial localization, dopamine receptor type, and embedding within specific circuitry (Ray et al., 2022; Chen et al., 2023; Nishioka et al., 2023). It has been proposed that neurons in the core and shell regions process different information in reward-related behaviors (Carelli, 2004; West and Carelli, 2016). By examining immediate early gene c-Fos expression in the NAc, studies indicate that although rewarding stimuli predominantly activate D1-MSNs, aversive stimuli could activate both D1- and D2-MSNs (Xiu et al., 2014; Sheng et al., 2023). *In vivo* recordings offer enhanced insights into the behavioral correlations with neural activity. For example, fiber photometry recording has revealed that D1- and D2-MSNs are both recruited during hedonic feeding, but represent different aspects of the food approach (Guillaumin et al., 2023). Analysis of single-cell calcium activity has revealed both types of MSNs exhibit diverse responses toward sucrose reward (Pedersen et al., 2022). D1- and D2-MSNs receive similar excitatory inputs and can interact in complex ways (Burke et al., 2017; Li et al., 2018; Keyes et al., 2020). MSNs are interconnected via lateral inhibition (Tunstall et al., 2002; Taverna et al., 2004), which is modulated by dopamine and serotonin (Dobbs et al., 2016; Burke and Alvarez, 2022). In addition, the D1-MSNs express dynorphin and substance P, while the D2-MSNs express adenosine 2A receptors (A2A) and enkephalin (Anderson and Reiner, 1990; Besson et al., 1990). A recent study revealed that endogenous enkephalin could suppress MSN activity via mu opioid receptors, which is necessary for potentiating reward consumption (Castro et al., 2021). D1- and D2-MSNs can regulate the function of interneurons by releasing neuropeptides, which in turn modulate neighboring MSN activity (Francis et al., 2019). D1-MSN stimulations can lead to variable behavioral outcomes (Table 1). Moreover, optogenetic activation of D2-MSN subsets has been shown to increase motivation (Soares-Cunha et al., 2016a, 2018; Yao et al., 2021), while inhibition of D2-MSNs in the core region could slow associative learning (Zachry et al., 2024). Thus, the activity dynamics and function of D2-MSNs are not simply the inverse of those of D1-MSNs, instead, recent evidence suggests D1- and D2-MSNs work in tandem.

**Table 1:** NAc pathways involved in the regulation of reward and aversion.

A. Input pathways					
Pathways	Subregion/cell types	Manipulations	Reward	Aversion	References
BLA ➔ NAc	BLA ➔ NAc	oActivation	oSS		Stuber et al. (2011), Britt et al. (2012), Namburi et al. (2015)
	rBLA ➔ lNAcSh	oActivation		Sucrose seeking ↓	Wang et al. (2020)
	BLA: FEZF2 ➔ NAc	oActivation		RTPA	Zhang et al. (2021)
	BLA: CCK^+^ ➔ NAcC: D2	oActivation		oSDP, RTPA	Shen et al. (2019)
	BLA: CCK^−^ ➔ NAcC: D1		oSS, RTPP		
	BLA ➔ NAc: FSI	oLTP	Cocaine SA ↑		Yu et al. (2017)
DR ➔ NAc	DR: 5-HT ➔ NAc	oActivation	N	N	Walsh et al. (2018) and Browne et al. (2019)
	DR: 5-HT ➔ NAc	oActivation	Forced swimming immobility ↓		Fontaine et al. (2022)
	DR: GABA ➔ NAc	pSilencing		Cocaine sensitization ↓	Kasper et al. (2016)
	DR: TH ➔ mNAcSh	oInhibition		SI ↓	Choi et al. (2022)
dHipp ➔ NAc	dHipp ➔ dmNAc	oActivation	oSS		Ibrahim et al. (2024)
	dHipp➔NAc	oInhibition		Spatial reward memories ↓	Barnstedt et al. (2024)
vHipp ➔ NAc	vHipp ➔ NAc	oActivation	oSS		Britt et al. (2012)
	vHipp ➔ NAc	oLTD	SP ↑, Forced swimming immobility ↓		Pignatelli et al. (2021)
	vCA1 ➔ NAcC: D1	pActivation	Cocaine CPP ↑		He G. et al. (2023)
	vCA1 ➔ NAcC: D2	pActivation		Cocaine CPP ↓	
PVT ➔ NAc	pPVT ➔ NAc	oActivation		RTPA, FC ↑	Zhu et al. (2016) and Beas et al. (2018)
	aPVT ➔ NAc	oActivation	Hedonic feeding ↑		Christoffel et al. (2021a)
	PVT: CRF ➔ NAc	oActivation		oSDP, RTPA	Engelke et al. (2021)
PFC ➔ NAc	PFC ➔ NAc	oActivation	oSS		Britt et al. (2012)
	PFC ➔ NAc	oActivation		Hedonic feeding ↓	Liu et al. (2016) and Christoffel et al. (2021a)
VP ➔ NAc	VP ➔ mNAcSh	oActivation	Hedonic feeding ↑		Vachez et al. (2021)
VTA ➔ NAc	VTA: DA ➔ vmNAcSh	–		FP during AL	de Jong et al. (2019)
	VTA: DA ➔ lNAcSh	–	FP during AL		
	VTA: DA ➔ vmNAcSh	oActivation	RTPP		Dong et al. (2023)
		oInhibition		Restrain-relief CPP ↓	
	VTA: DA ➔ lNAcSh	oActivation	RTPP		
		oInhibition		Restrain-relief CPP ↓	
	VTA: GABA ➔ NAc	oActivation	N	N	van Zessen et al. (2012)
		pActivation	N	N	Wakabayashi et al. (2019)
	VTA: GABA ➔ NAcSh: CIN	oActivation	oSS, RTPP, AL		Brown et al. (2012) and Al-Hasani et al. (2021)
	VTA: VGLUT2 ➔ NAc	oActivation+sgTH	oSS		Warlow et al. (2024)
	VTA: VGLUT2 ➔ NAc	oActivation+sgVGLUT2		RTPA	
	VTA: VGLUT2 ➔ NAc: FSI	oActivation		RTPA	Qi et al. (2016)

B. Output pathways					
Pathways	Subregion/cell types	Manipulations	Reward	Aversion	References
NAc ➔ IPAC	NAcSh: D1 ➔ mIPAC: GABA	pActivation	CPP ↑		Liu et al. (2023)
NAc ➔ LH	NAcC: D1 ➔ LH: GABA	pSilencing	CPA ↓		Sheng et al. (2023)
	mNAcSh: TAC1 ➔ LH	oInhibition		Avoidance ↑	He Z. X. et al. (2023)
	mNAcSh: TAC2 ➔ LH	oActivation		Cocaine CPP ↓	Zhao et al. (2022)
	^BLA➔^NAc ➔ LH	oActivation	oSS, RTPP		Zhou et al. (2022)
	^PVT➔^NAc ➔ LH	oActivation		RTPA, oSDP	
NAc ➔ VP	NAc: D1 ➔ VP	oActivation		RTPA	Liu et al. (2022)
	NAc: D2 ➔ VP	oActivation		RTPA	
	dmNAcSh: D2 ➔ VP	oActivation	oSS, RTPP		Yao et al. (2021)
	vmNAcSh/vlNAcSh: D2 ➔ VP	oActivation		RTPA	
	lNAcSh: TAC1 ➔ VP	pActivation	SI ↑, SP ↑		He et al. (2020)
		pSilencing		SI ↓, SP ↓	
	NAc: D2 ➔ VP	oActivation	Responses ↑ during Al: cue	Responses ↓ AI: reward	Soares-Cunha et al. (2022)
NAc ➔ VTA	mNAcSh ➔ VTA	oActivation	Hedonic feeding ↓		Bond et al. (2020)
		oInhibition		Hedonic feeding ↑	
	NAc: D1 ➔ VTA: GABA	oActivation	oSS, RTPP		Edwards et al. (2017), Liu et al. (2022), and Zhou et al. (2022)
	mNAcSh ➔ VTA: DA	oActivation		RTPA	Yang et al. (2018) and Chen et al. (2023)
	lNAcSh ➔ VTA: GABA	oActivation	oSS, RTPP		

5-HT, 5-hydroxytryptamine; AL, associative learning; aPVT, anterior paraventricular thalamus; BLA, basolateral amygdala; CCK, cholecystokinin; CIN, cholinergic interneuron; CPP, conditioned place preference; CRF, corticotropin releasing factor; D1, D1 type dopamine receptor; D2, D2 type dopamine receptor; DA, dopamine; DAT, dopamine transporter; dmNAcSh, dorsomedial shell of the nucleus accumbens; DR, dorsal raphe; FC, fear conditioning; FEZF2, forebrain expressed zinc finger 2; FP, fiber photometry; GABA, gamma-aminobutyric acid; lNAcSh, lateral shell of the nucleus accumbens; IPAC, interstitial nucleus of the posterior limb of the anterior commissure; LH, lateral hypothalamus; N, no effect; NAc, nucleus accumbens; NAcC, core region of the NAc; NAcSh, shell region of the NAc; oActivation, optogenetic activation; oInhibition, optogenetic inhibition; oLTP, optogenetic induced long-term potentiation; oLTD, optogenetic induced long-term depression; oSDP, optic stimulation during positive reinforcement; oSS, optic self-stimulation; pActivation, pharmacological activation; PFC, prefrontal cortex; PIT, Pavlovian-instrumental transfer; pPVT, posterior paraventricular thalamus; pSilencing, pharmacological silencing; PV, parvalbumin; PVT, paraventricular thalamus; rBLA, rostral basolateral amygdala; RTPA, real-time place aversion; RTPP, real-time place preference; SA, self-administration; SI, social interaction; SP, sucrose preference; TAC1, tachykinin precursor 1; TH, Tyrosine hydroxylase; vCA1, ventral CA1 region of the hippocampus; VGLUT2, vesicular glutamate transporter 2; vHipp, ventral hippocampus; vlNAcSh, ventrolateral shell of the nucleus accumbens; vmNAcSh, ventromedial shell of the nucleus accumbens; VP, ventral pallidum; VTA, ventral tegmental area.

Cholinergic interneurons (ClNs) account for approximately 1–2% of NAc neurons (Zhou et al., 2002; Witten et al., 2010) and are more densely populated in the shell region than the core (Meredith et al., 1989). They receive inhibitory inputs from neighboring interneurons and MSNs, as well as GABAergic neurons in the VTA (Brown et al., 2012; Al-Hasani et al., 2021; Baimel et al., 2022). ClNs are tonically active, providing the main source of acetylcholine (ACh) in the NAc (Aosaki et al., 1995; Zhou et al., 2002). Neighboring CINs often show a synchronized cessation of firing in response to reward or associated cues (Goldberg and Reynolds, 2011; Marche et al., 2017), paralleled by a deflect in ACh release (Skirzewski et al., 2022), which might be regulated by dopaminergic and glutamatergic inputs (Chantranupong et al., 2023). On the contrary, an increase of ACh in the NAc is often associated with aversive stimuli or satiety (Helm et al., 2003; Rada et al., 2004; Hoebel et al., 2007; Avena and Rada, 2012). Recent studies have shown that activation of ClNs attenuates conditioning responses to rewards (Collins et al., 2019; Gallo et al., 2022), whereas their inhibition seems to enhance reward reinforcement (Al-Hasani et al., 2021). Although CINs predominantly target MSNs, they also form synapses with other interneurons (English et al., 2011; Mamaligas and Ford, 2016). ACh may modulate neuronal activity directly through muscarinic and nicotinic cholinergic receptors located postsynaptically (Collins et al., 2016; Mamaligas and Ford, 2016), and is also implicated in the regulation of neurotransmitter release (Abudukeyoumu et al., 2019; Reynolds et al., 2022), including dopamine (Cachope et al., 2012; Mohebi et al., 2023). Nonetheless, the influence of ACh on these processes can vary, influenced by the specific subregion of the NAc and receptor types involved (Collins et al., 2016; Shin et al., 2017; Klawonn et al., 2018; Mancini et al., 2022).

The GABAergic interneurons in the NAc are a heterogeneous assembly, comprising fast-spiking interneurons (FSIs), low-threshold-spiking interneurons (LTSI), and neurogliaform cells (NGC) (Tepper et al., 2018). These interneurons are recipients of the same excitatory inputs as the MSNs and contribute to the feedforward inhibition of the MSNs (Qi et al., 2016; Scudder et al., 2018; Trouche et al., 2019). FSIs, identified by their expression of the calcium-binding protein parvalbumin (Bennett and Bolam, 1994), are primarily responsible for fast inhibitory neurotransmission (Wright et al., 2017). Synchronized activation of FSIs promotes conditioned aversion (Schall et al., 2021; Xiao et al., 2021), while certain FSI subpopulations are essential for reward memory retrieval (Trouche et al., 2019), suggesting a nuanced functional diversity among these neurons. LTSIs and NGCs, characterized by their expression of the neuropeptide somatostatin (Kawaguchi et al., 1995) and neuropeptide Y (NPY) (Ibanez-Sandoval et al., 2011) respectively, modulate MSN activity in a more sustained manner (Tepper and Bolam, 2004). Recent studies have shown that LTSIs undergo contrasting changes in excitability following cocaine exposure and footshock experiences (Ribeiro et al., 2018; Kondev et al., 2023). However, how LTSIs participate in valence encoding is still not clear. The involvement of NGCs in reward-related behaviors warrants further investigation.

## 4 NAc pathways involved in reward and aversion processing

The NAc receives glutamatergic inputs from limbic and cortical areas like the prefrontal cortex (PFC), basolateral amygdala (BLA), ventral hippocampus (vHipp), thalamus (Li and Kirouac, 2008), and is reciprocally connected with the ventral tegmental area (VTA), ventral pallidum (VP), and lateral hypothalamus (LH) (Haber and Knutson, 2010). Studies using trans-synaptic viral strategy and optogenetics combined with patch-clamp electrophysiology have revealed diverse monosynaptic connectivity patterns of neurons in the NAc (Ma et al., 2020; Yao et al., 2021). In general, neurons in different subregions of the NAc exhibit distinct profiles of afferent and efferent connections (Groenewegen et al., 1999). For instance, projections from the vHipp mainly terminate within the medial NAcSh (Kelley and Domesick, 1982). On the other hand, projections from the dorsal prelimbic area predominantly terminate within the core region (Berendse et al., 1992a). Notwithstanding, within a given subregion, D1- and D2-MSNs tend to receive similar inputs (Barrientos et al., 2018; Li et al., 2018). The medial and lateral shell of the NAc targets the medial and lateral parts of the VTA, respectively (Lammel et al., 2012), while the NAcC innervates the substantia nigra (Berendse et al., 1992b). The projections from NAc subregions to the VP and LH are also topographically arranged (Heimer et al., 1991). Although both D1 and D2-MSNs project to the VP, the majority of neurons targeting the VTA and LH express the D1 receptor (Kupchik et al., 2015; Thoeni et al., 2020). Notably, subpopulations of D1-MSNs synapse on GABAergic interneurons in the downstream regions, which produce motivational drive via disinhibition (Watabe-Uchida et al., 2012; Yang et al., 2018; Thoeni et al., 2020; Yao et al., 2021).

Considering the diversity of inputs, MSNs could process and relay distinct pieces of information to specific downstream neurons, thereby orchestrating different motivational states (LeGates et al., 2018; Tong et al., 2024). The PFC inputs to the NAc convey cognitive and executive control signals, influencing decision-making processes and goal-directed behavior (Sun and Rebec, 2006; Domingo-Rodriguez et al., 2020). The input from the vHipp contributes spatial and contextual information to the NAc, influencing memory-related processes and spatial navigation, which may guide goal-directed behaviors in various environmental contexts (Pennartz et al., 2011). The amygdala relays emotional information to the NAc, modulating cue-triggered motivated behaviors (Ambroggi et al., 2008; Stuber et al., 2011). Manipulations of distinct inputs of the NAc might have opposing effects on reward-related behaviors. For instance, activations of the BLA ➔ NAc and PVT ➔ NAc pathways have been found to induce reward and aversion, respectively (Stuber et al., 2011; Zhu et al., 2016). It has been suggested that the BLA and PVT might target different MSN ensembles, which in turn exhibit distinct projection patterns. The MSNs receiving BLA input preferentially target GABAergic neurons in the VTA and glutamatergic neurons in the LH to mediate reward, whereas MSNs receiving PVT input are associated with aversive outcomes, primarily through the inhibition of GABAergic neurons in the LH (Zhou et al., 2022). Interestingly, it has been revealed that both the BLA and PVT encompass various neuron subtypes and functional subregions that might mediate opposing valence (Namburi et al., 2015; Shen et al., 2019; Christoffel et al., 2021a; Poggi et al., 2024). Both inputs innervate interneurons in the NAc (Yu et al., 2017; Paniccia et al., 2024), in addition to targeting directly on MSNs. Thus, convergence and segregation of heterogenous inputs might modulate MSN activity and contribute to the processing of reward and aversion in a more dynamic and complex way than previously expected. The recent discoveries utilizing optogenetic or pharmacogenetic approaches to dissect the functionality of NAc pathways are summarized in Table 1.

## 5 Monoamine neurotransmitters modulate NAc function

Multiple monoamine neurotransmitters modulate the activity of NAc neurons. These neurotransmitters transmit crucial information about rewards, punishment, and arousal, indispensable for adaptive motivation and learning (Castro and Bruchas, 2019). The following subsections will delve into the properties of dopamine, serotonin, and norepinephrine signals within the NAc, addressing the origin of their inputs, the stimuli or conditions that induce their release, the receptor types involved, and the behavioral relevance of these processes.

### 5.1 Dopamine (DA)

Dense dopaminergic projections from the ventral tegmental area (VTA) target the NAc, establishing a critical neurotransmitter for reward in this region (Schultz, 1998; Bjorklund and Dunnett, 2007; Haber and Knutson, 2010). DA neurons in the VTA exhibit irregular firing patterns with phasic high frequencies of action potentials (Marinelli and McCutcheon, 2014; Berke, 2018), potentially instigating transient surges in DA release (Floresco et al., 2003; Liu et al., 2018). Additionally, DA fluctuations in the NAc are subject to regulation by spike-independent processes regulating DA release and uptake (Mohebi et al., 2019; Morrens et al., 2020; Sabandal et al., 2021). For instance, presynaptic opioid and nicotinic receptors could modulate DA release in the NAc (Britt and McGehee, 2008; McGovern et al., 2023). DA concentration in the NAc is also regulated by glutamatergic inputs from the BLA and ACh release from local CINs (Floresco et al., 1998; Jones et al., 2010; Threlfell et al., 2012; de Jong et al., 2022). DA neurons display heterogeneity in their activity, co-released transmitters, and axon projections (Morales and Margolis, 2017). DA neurons in the VTA can be activated by both rewarding and aversive stimuli (Matsumoto and Hikosaka, 2009), yet it is believed that DA projections to the NAc primarily signal reward-related events (Day et al., 2007; Lammel et al., 2008, 2011; Gunaydin et al., 2014). Stimulation of DA terminals in the NAc is sufficient to support self-stimulation or induce place preference, implying that dopamine release *per se* can act as reinforcement (Tsai et al., 2009; Witten et al., 2011; Jing et al., 2022). Conversely, depletion of DA with 6-OHDA in the NAc impairs not only reward seeking but also punishment avoidance (Bergamini et al., 2016). Utilizing genetically encoded sensors, recent research has revealed the diversity of dopamine signaling throughout the NAc (Patriarchi et al., 2018; Sun et al., 2018). While reward stimuli increase DA release in most of the NAc, aversive stimuli specifically augment DA levels in the ventromedial shell of the NAc (vmNAcSh) (de Jong et al., 2019; Yuan et al., 2019). In addition, the cession of the punishment servers as a relief reward to increase DA release in the dorsomedial shell and the lateral shell of the NAc (dmNAcSh and lNAcSh) (Dong et al., 2023). Silencing the DA input to the NAc shell abolishes relief learning (Mayer et al., 2018; Dong et al., 2023). In NAcC, dopamine release denotes perceived saliency (Kutlu et al., 2021; Goedhoop et al., 2022; Jeong et al., 2022) rather than adhering to a prediction error model (Bayer and Glimcher, 2005; Schultz, 2016). A recent study indicates that optogenetic manipulation of the DA response to a novel stimulus in the NAcC, prior to training, can bidirectionally influence the stimulus’s capacity to act as a predictive cue for punishment (Kutlu et al., 2022), highlighting the significance of DA in novelty-based learning within this subregion.

Dopamine receptors are widely distributed across the NAc, existing in both pre-and postsynaptic locations (Goto and Grace, 2005). D1-like receptors (D1 and D5) and D2-like receptors (D2, D3, and D4) are coupled to Gs/olf and Gi/o signaling pathways, respectively, modulating neuronal excitability and synaptic plasticity in distinct manners (Soares-Cunha et al., 2016b; Lee et al., 2021; Tsuboi et al., 2022). For example, D1 receptors may promote the phosphorylation of AMPA receptors and increase spine density, thereby intensifying behavioral responses to drug rewards (Hobson et al., 2013; Nagai et al., 2016). The extinction of drug memory is associated with Rac1-dependent reduction in spine density in D1-MSNs (Tu et al., 2019; Zhao et al., 2019), which could be attenuated by D1 receptor stimulation (Kobrin et al., 2017). In addition, D1R in different NAc subregions might contribute to distinct aspects of reward processing (Gore and Zweifel, 2013; Hauser et al., 2015). Notably, evidence also points to D1 receptors playing a role in aversion processing (Acquas and Di Chiara, 1994; Chartoff et al., 2006; Kim et al., 2007), supporting the multifaceted role of DA transmission in the NAc (Verharen et al., 2020). D2-like receptors are also indispensable for reward and reward association (Caine et al., 2002; Tran et al., 2002). Knockdown of D2-like receptors in the NAc attenuates drug reward (Miyamoto et al., 2014), while their overexpression enhances motivation (Trifilieff et al., 2013), probably by suppressing inhibitory transmission to the VP (Gallo et al., 2018). It has thus been proposed that D1- and D2-like receptors play a cooperative role in regulating reward-related behaviors (Self and Stein, 1992; Ikemoto et al., 1997; Schmidt and Pierce, 2006; Steinberg et al., 2014). Compared with D1 receptors, D2 receptors have a higher affinity for DA (Richfield et al., 1989; Beaulieu and Gainetdinov, 2011). This characteristic potentially allows D2 receptors to detect dips in DA release (Iino et al., 2020), which might represent a negative prediction error (Chang et al., 2016), thereby mediating aversion (Danjo et al., 2014) and enabling reward discrimination (Iino et al., 2020; Nishioka et al., 2023). However, caution is warranted when interpreting data from studies involving drugs of abuse, where excessive DA release could lead to aberrant activation of receptors, which does not occur with natural rewards. In addition, hedonic states do not rely exclusively on dopamine receptors (Narayanan et al., 2004).

In summary, the role of D1 and D2 receptors in the NAc is not simply promoting reward and aversion, respectively. The information transmitted through the DA signal to the NAc may vary depending on behavioral context and should be analyzed with cell-type and pathway-specific evaluations in future studies. Additionally, a small proportion of MSNs co-express both D1 and D2 receptors, the functional significance of which remains to be elucidated.

### 5.2 Serotonin (5-hydroxytryptamine or 5-HT)

Serotoninergic innervation of the NAc originates primarily from the dorsal raphe nucleus (DRN) and, to a lesser extent, from the median raphe nucleus (MRN) (Vertes and Martin, 1988; Ren et al., 2018). 5-HT terminals establish synaptic contacts with both dendrites and axon terminals in the NAc (Van Bockstaele and Pickel, 1993), influencing NAc activity via a range of presynaptic and postsynaptic mechanisms (Hayes and Greenshaw, 2011; Virk et al., 2016; Christoffel et al., 2021a). 5-HT neurons in the DR show diverse response patterns toward a variety of rewarding and aversive stimuli (Liu et al., 2014; Li et al., 2016; Okaty et al., 2019). The 5-HT signal in the NAc has been associated with sociability and drug reward (Liu Z. et al., 2020). Optogenetic stimulation of the DR ➔ NAc pathway or inhibition of 5-HT re-uptake within the NAc enhances preferences for non-aggressive social interactions, without directly reinforcing behaviors (Walsh et al., 2018). The prosocial effect of 5-HT is dependent on the 5-HT1b receptors located predominantly at presynaptic sites in the NAc (Dolen et al., 2013; Pomrenze et al., 2022). It has been demonstrated that 5-HT depresses excitatory synaptic transmission to the NAc in an input-specific way (Christoffel et al., 2021a). Emphathogens such as MDMA are known to engender prosocial effects, partly through the inhibition of 5-HT re-uptake (Heifets et al., 2019). A recent study suggests that the 5-HT2a receptor is implicated in the reopening of the critical period for social reward learning, induced by lysergic acid diethylamide (LSD) and psilocybin (Nardou et al., 2023). However, ketamine and MDMA induce reinstatement of social reward learning in a 5-HT2a-independent way. There are conflicting reports regarding the role of 5-HT in mediating drug reinforcement effects (Roger-Sanchez et al., 2013; Matuskey et al., 2014; Heifets et al., 2019). Nevertheless, studies indicate that the 5-HT1b receptor modulates the rewarding effect of cocaine in a context-dependent manner (Barot et al., 2007; Pentkowski et al., 2012, 2014) and plays an important role in developing compulsive drug-seeking behavior (Li et al., 2021). Activation of 5-HT6 receptors in the NAc has been shown to promote both natural and drug reward (Ferguson et al., 2008; Pratt et al., 2012). Interestingly, despite both NAc and 5-HT signaling being associated with the suppression of premature reward-seeking behaviors (Bouwknecht et al., 2001; Basar et al., 2010; Miyazaki et al., 2014, 2018), direct stimulation of 5-HT terminals or pharmacological activation of the 5-HT2c receptor in the NAc does not promote patience for reward waiting (Miyazaki et al., 2020; Harmson et al., 2022).

### 5.3 Norepinephrine

Norepinephrine is another monoamine neurotransmitter that innervates the NAc, with primary projections arising from the locus coeruleus (LC) and the nucleus of the solitary tract (NST) (Delfs et al., 1998). The activity of catecholaminergic neurons in the LC and NST is typically suppressed during reward consumption (Roberts et al., 2017; Sciolino et al., 2022). NE signaling plays a key role in the regulation of arousal and the assignment of salience to stimuli (Ventura et al., 2007). NE release in the NAc is increased in response to aversive stimuli (Gomez-Milanes et al., 2012), and the direct administration of NE into the NAc produces a stimulatory effect (Svensson and Ahlenius, 1982; Plaznik et al., 1985). NE mediates its effect mainly through α- and β-adrenergic receptors, which are located in both dendrites and axons in the NAc (Mitrano et al., 2012). As a result, NE plays an important role in regulating glutamatergic and dopaminergic transmission in the NAc (Aono et al., 2007; Saigusa et al., 2012; Peng et al., 2018). Notably, NE signaling has been shown to selectively influence glutamatergic synapses onto FS interneurons, with minimal direct impact on MSNs (Manz et al., 2021). Although recording NE signals in freely moving animals has been challenging, there have been recent advances in the development of genetically encoded NE sensors (Feng et al., 2019; Kagiampaki et al., 2023; Mao et al., 2023). Monitoring and manipulating NE release in the NAc with high temporal and spatial precision during approach and avoidance behaviors presents a significant opportunity for future research.

The DA, 5-HT, and NE systems do not act in isolation but rather interact to finely tune the NAc’s response to environmental stimuli. In addition to the coordination of neuronal activity in regions upstream of the NAc, monoamines also locally interact within the NAc to modulate reward processing. On one hand, monoamine receptors, extensively located on presynaptic terminals within the NAc, modulate the release of various neurotransmitters. For instance, adrenoceptors may play an inhibitory role in DA efflux (Kochenborger et al., 2012), potentially contributing to an anhedonic state. On the other hand, DA and NE share structural similarities and can bind to each other’s receptors and transporters (Borgkvist et al., 2012). As a result, 6-OHDA can be taken up by both the dopamine transporter (DAT) and the norepinephrine transporter (NET), subsequently inducing damage to both DA and NE terminals (Braun et al., 2016). Additionally, 5-HT can be transported by the DAT and subsequently be released together with DA (Stamford et al., 1990; Zhou et al., 2005), and the 5-HT transporter is also capable of transporting DA (Kannari et al., 2006; Larsen et al., 2011). While the crosstalk between monoaminergic systems may not be evident under normal physiological conditions, it should not be overlooked in drug studies (Hall et al., 2004). Notably, the distribution and functionality of these neurotransmitters vary within different regions of the NAc (Pickel and Chan, 1999; Brown and Molliver, 2000; Chuhma et al., 2014), which could result in inconsistencies in the outcomes of pharmacological interventions. Addressing these nuances remains an important challenge for future research.

## 6 Role of the NAc in drug addiction

Drug addiction, a chronic and relapsing brain disorder, is characterized by compulsive drug seeking and use, loss of control over intake, and negative emotional states during withdrawal (Koob and Volkow, 2016). The NAc is a central hub in the neural circuitry of addiction. Studies have shown that animals will self-administer (SA) a variety of drugs directly into the NAc (McBride et al., 1999). Diverse genetically defined or pathway-specific neuron ensembles in the NAc contribute to distinct behavioral and emotional aspects of drug addiction (Luscher and Malenka, 2011; Zinsmaier et al., 2022). The NAc responds to both addictive drugs and environmental cues associated with drug use (Table 2). Drugs of abuse influence NAc neuronal activity in multiple ways, including alterations in gene expression, direct receptor activation, modulation of DA release, and changes in synaptic transmission (Dobbs et al., 2016; Christoffel et al., 2021b; Teague and Nestler, 2022; He Y. et al., 2023).

**Table 2:** *In vivo* monitoring of neuronal activity and neurotransmitter release in the NAc during reward-related behaviors.

A. Calcium activity						
Contents	Approaches	Regions/neurons	Rewards	Administrations/behaviors	Response	References
Calcium	Fiber photometry	NAc	HF food	Oral intake	↑	Wu et al. (2022)
		NAc: D1			↑	
		NAc: D2			N	
		NAc: D1	Cocaine-paired context	Cocaine-CPP test	↑	Calipari et al. (2016)
		NAc: D2			↓	
		NAc: D1	Propofol	Acute i.p. injection	↑	Zhu X. N. et al. (2023)
		NAc: D2			↓	
		NAc: D1	Social	Social interaction	↑↓	Zhao et al. (2023)
		NAc: D2			↑↓	
		NAc: CIN	Food	Oral intake	↓	Al-Hasani et al. (2021)
		NAc: PV	Food	Oral intake	↑	Pisansky et al. (2019)
		NAc^➔VP^	Heroin-paired context	Heroin-CPP test	↓	O'Neal et al. (2022)
		NAc^➔VTA^			↑	
		mNAc^➔VTA^	Food	Oral intake	↓	Bond et al. (2020)
	Calcium imaging	NAc: D1	Cocaine	Acute i.p. injection	↑↓	van Zessen et al. (2021)
		NAc: D2			↑↓	
		mNAcSh: Pdyn	Sucrose/water	Oral intake	↑↓	Pedersen et al. (2022)
		mNAcSh: Penk			↑↓	
		mNAcSh: Penk	Food	Oral intake following food deprivation	↑↓	Castro et al. (2021)
		mNAcSh	Water	Oral intake	↑↓	Chen et al. (2023)
		lNAcSh			↑↓	
		NAcC			↑↓	

B. Neurotransmitter release						
Contents	Approaches	Regions/neurons	Rewards	Administrations/behaviors	Response	References
5-HT	Microdialysis	NAc	Cocaine/METH	Acute i.p. injection	↑	Reith et al. (1997) and Ruda-Kucerova et al. (2015)
	Voltammetry	NAc	Cocaine	Acute i.p. injection	↑	Broderick et al. (2003)
	Fiber photometry	mNAcSh	Social	Social interaction during morphine withdrawal	↑^R^	Pomrenze et al. (2022)
ACh	Microdialysis	NAc	Basal ACh	Sucrose withdrawal	↑	Avena et al. (2008)
			Food/water	At the end of a meal	↑	Mark et al. (1992) and Rada et al. (2005)
			Morphine	Acute i.p. injection	↓	Rada et al. (1991) and Rada et al. (1996)
				Precipitated withdrawal	↑	
			Nicotine	Acute s.c.injection	↑	Rada et al. (2001)
				Precipitated withdrawal	↑	
		NAcC	Cocaine	i.v. injection	↑	Crespo et al. (2006)
			Morphine	i.v. injection	↑	Crespo et al. (2008)
	Fiber photometry	NAc	Food	Oral intake	↓	Skirzewski et al. (2022)
		NAcC			↑ + ↓	Gallo et al. (2022)
		vmNAcSh			↓	Al-Hasani et al. (2021)
DA	Microdialysis	NAc	Alcohol/AMPH/Cocaine/Ketamine/MDMA	Acute i.p. injection	↑	Marinelli et al. (2003), Masuzawa et al. (2003), McKittrick and Abercrombie (2007), Tourino et al. (2008), and Xi et al. (2011)
			Cannabinoid/Cocaine/Heroin/MDMA/METH/Nicotine	i.v. injection	↑	Pontieri et al. (1996), Tanda et al. (1997), Trigo et al. (2007), Ferris et al. (2011), and Zhang et al. (2018)
			Sex	Sexual interaction/female urine sniffing test	↑	Malkesman et al. (2010) and Canseco-Alba et al. (2022)
			Food/ethonal	Oral intake	↑	Sokolowski et al. (1998), Di Chiara et al. (1999), and Hamid et al. (2016)
			Nicotine	Re-exposure during withdrawal	↑^I^	Zhang et al. (2012)
			Basal DA	During nicotine withdrawal	↓	
	Voltammetry	NAc	Cocaine	Acute i.p. injection	↑	Broderick et al. (2003)
			Food	Oral intake	↑	Hamid et al. (2016)
		NAcC	Nicotine	Acute i.p. injection	N	Nisell et al. (1997)
		NAcSh			↑	
		NAcC	Cocaine	Acute i.p. injection	↑	Congestri et al. (2008)
		NAcSh				
	Fiber photometry	mNAcSh	Alcohol	Oral intake	↓	Liu Y. et al. (2020)
			AMPH/heroin/propofol	Acute i.p. injection	↑	Corre et al. (2018), Terauchi et al. (2023), and Zhu et al. (2023a)
			Heroin-paired context	Heroin-CPP test	↑	O'Neal et al. (2022)
			Social	Social interaction following isolation	↑	Choi et al. (2022)
			Strawberry milkshake	Oral intake	↑	Skirzewski et al. (2022)
		dmNAcSh	Stress relief	Relief from restrain	↑	Dong et al. (2023)
		lNAcSh	Alcohol	Oral intake	↑	Liu Y. et al. (2020)
			Cocaine	i.v. injection	↑	Pribiag et al. (2021)
			Food/sucrose water	Oral intake	↑	Yuan et al. (2019) and Mazzone et al. (2020)
			Stress relief	Relief from restrain	↑	Dong et al. (2023)
		NAcC	Alcohol	Oral intake	↑	Liu Y. et al. (2020)
			Cocaine	i.v. injection	↑	Lujan et al. (2023)
			Fentanyl	Acute i.p. injection	↑	Lefevre et al. (2020)
				Acute i.p. injection after interrupted morphine exposure	↑^I^	
			Social	Approach, investigation, and action phases of social behaviors	↑	Dai et al. (2022)
NE	Microdialysis	NAc	Alcohol	Acute i.p. injection	N	Marinelli et al. (2003) and Karkhanis et al. (2014)
				Acute i.p. injection during social isolation	↑	Karkhanis et al. (2014)
			AMPH/cocaine	Acute i.p. injection	↑	Reith et al. (1997) and McKittrick and Abercrombie (2007)
			Sucrose	Sham intake orosensory only	↓	Hajnal and Norgren (2004)

AMPH, amphetamine; CPP, conditioned place preference; MDMA, 3–4 methylenedioxymethamphetamine; METH, methamphetamine; N, no effect; ↑, increased activity or release of neurotransmitter; ↓, decreased activity or release of neurotransmitter; ↑^R^, reduced release compared to baseline; ↑^I^, increased release compared to baseline; ↑↓, a higher percentage of excited neurons than inhibited neurons; ↑↓, a higher percentage of inhibited neurons than excited neurons; ↑↓, heterogeneous responses with similar amount of neurons showing excitatory and inhibitory response; ↑ + ↓, a peak followed by a dip.

The DA system plays a pivotal role in mediating the rewarding or reinforcing effects of addictive substances (Luscher and Pascoli, 2021; Samaha et al., 2021). Most drugs of abuse enhance DA release in the NAc (Di Chiara and Imperato, 1986; Johnson and North, 1992; Morel et al., 2019; Simmler et al., 2022) via a variety of mechanisms, such as stimulating or disinhibiting DA neurons, augmenting DA release, and blocking DA re-uptake (Chen et al., 2006; Venton et al., 2006; Sulzer, 2011; Bocklisch et al., 2013). The DA release in the NAc, particularly in the shell region, correlates with the initial rewarding effect of drug use (Di Chiara, 2002; Calabrese, 2008; Ito and Hayen, 2011; Floresco et al., 2018). Evidence suggests that the accumbal D1 receptor is essential for the positive reinforcing effects of drugs of abuse (Caine et al., 2007; Chellian et al., 2022). The DA responses to ongoing drug use vary with the drug intake regimen (Ferris et al., 2012; Calipari et al., 2013). With sporadic drug exposure, the DA response to drugs or associated cues may escalate over time (Addy et al., 2010), which could fortify drug memories and eventually promote drug- or cue-induced relapse (Bossert et al., 2013; Jing et al., 2022). Conversely, this process may hinder the learning and memory associated with natural rewards (Luscher and Malenka, 2011; Saddoris et al., 2017). During periods of drug abstinence, an anhedonia emotional state has been linked to reduced basal DA levels (Volkow et al., 2007). Interestingly, D1 and D2 receptor-expressing MSNs are both involved in withdrawal symptoms (Fox et al., 2023), likely through different downstream pathways. Apart from DA, a spectrum of neurotransmitter systems within the NAc are implicated in addiction. For example, the 5-HT release in the NAc increases following drug taking and decreases during withdrawal (Weiss et al., 1996; Broderick et al., 2004), contributing to the hedonic and anhedonic states experienced in addiction. Neuropeptide Y (NPY) in the NAc has been shown to potentiate drug reward (Tanaka et al., 2021). ACh, dynorphin, and norepinephrine signals in the NAc all play a part in the aversion associated with withdrawal (Mu et al., 2011; Almela et al., 2012; Gomez-Milanes et al., 2012).

Prolonged exposure to addictive substances induces enduring changes in neural plasticity within the NAc, which are crucial for the development of persistent drug-related memories and atypical behaviors (Parsegian and See, 2014). Following drug exposure, there is evidence of *de novo* spine formation in MSNs, which could be mediated by brain-derived neurotrophic factor (BDNF) signaling (Huang et al., 2009; Barrientos et al., 2018). The regulation of the amount and function of glutamatergic receptors and transporters has also been observed during the processes of drug-seeking and drug-memory formation (Lu et al., 2004; Conrad et al., 2008; Loweth et al., 2014; Niedzielska-Andres et al., 2021), whereas different drugs produce cell-specific effects on spines and synapses in the NAc (Graziane et al., 2016). Repeated drug use also alters GABA transmission in the NAc (Xi et al., 2003). Furthermore, changes in the intrinsic excitability of MSNs following drug use have been reported (Mu et al., 2010; He Y. et al., 2023). These structural and electrophysiological changes within the NAc may vary depending on the cell type and source of synaptic input (Thompson et al., 2021; Zhao et al., 2022; Zinsmaier et al., 2022). For instance, the BLA neurons, encoding emotional valence, might fine-tune the activities of MSNs for the acquisition of operant behavior and cue–reward association (Ambroggi et al., 2008; Yu et al., 2017). The maturation of silent synapses of the BLA ➔ NAc pathway during abstinence has been associated with cocaine craving (Lee et al., 2013). Inputs from the vHipp to the NAc, providing spatial information, play a role in drug-related contextual memories (Pascoli et al., 2014; Zhou et al., 2019, 2020), and the transmission is potentiated following cocaine exposure (Britt et al., 2012). Drug-induced adaptations in the PVT ➔ NAc pathway have also been revealed, which might differently contribute to withdrawal symptoms and the reinstatement of drug-seeking behaviors (Zhu et al., 2016; Giannotti et al., 2021; Paniccia et al., 2024). On the contrary, cocaine-seeking behavior correlates with decreased synaptic transmission from the PFC (Pascoli et al., 2014), which exerts executive control (Koob and Volkow, 2010; Chen et al., 2013). Targeted reversal of these synaptic alterations can significantly modify the likelihood of drug relapse (Gipson et al., 2013; Heinsbroek et al., 2021). Specifically, the maturation of silent synapses on infralimbic ➔ NAc and prelimbic ➔ NAc pathways inhibits and promotes drug-seeking, respectively (Ma et al., 2014).

Overall, these maladaptive changes in the NAc can undermine the normal systems for motivation control and cognitive flexibility, resulting in cravings and compulsive drug-seeking behavior (Everitt and Robbins, 2005). Restoring control over such behavior could be achieved by counteracting the pathological effects of drug exposure (Kalivas and O'Brien, 2008; Moussawi et al., 2011). Pharmacological approaches aiming to replace or neutralize the effect of aberrant receptor activations in the NAc induced by drug taking or withdrawal have been proven to be effective (Nestler, 2005; Koob et al., 2009). Methadone, a long-acting agonist of opioid receptors, is commonly prescribed as a substitution treatment for drug detoxification (Leri et al., 2006; Bell and Strang, 2020). D3/D2 receptor partial agonists have shown potential to decrease psychostimulant intake (Neisewander et al., 2014). Approaches such as normalizing NAc activity with deep brain stimulation (DBS) and transcranial-focused ultrasound (tFUS) have also shown promise (Legon et al., 2014; Niu et al., 2020; Chang et al., 2022). The genetic and epigenetic changes that underpin drug-induced neuroplasticity within the NAc present new targets for developing innovative treatments for drug addiction (Ribeiro et al., 2018; Gallegos et al., 2022). Moreover, immunotherapy represents a novel and potentially transformative approach to restoring NAc function in individuals with substance abuse, as highlighted by recent research from Zhu et al. (2023b). These strategies may offer hope for more effective treatments that could address the underlying neurobiological changes associated with addiction.

## 7 NAc and reward processing deficits in depression

Depression, a widely prevalent mental health disorder, involves a complex interplay of genetic, environmental, and neurobiological factors (Duman et al., 2019; Ormel et al., 2019). Despite the inability of animal models to fully replicate the spectrum of human depression symptoms, they allow for the assessment of key elements such as anhedonia, passive coping, and social avoidance. Both in patients and animal models, these behavioral abnormalities have been linked to dysfunctions of the NAc (Pizzagalli et al., 2009; Wacker et al., 2009).

Chronic stress is a significant risk factor for mood disorders. It is frequently employed to study stress-related reward processing deficits in psychiatric disorders in animal research. Chronic stress induces profound changes in the structure and function of the NAc (Wook Koo et al., 2016; Qi et al., 2022). Extensive studies have been conducted to examine the morphological changes in dendrites and dendritic spines in the NAc (Qiao et al., 2016; Fox et al., 2020). NAc neurons exhibit changes in activity and plasticity in response to stress that are specific to the type of cell and neural pathways (Heshmati et al., 2020; Pignatelli et al., 2021). Moreover, stress alters the release of transmitters and neuromodulators in the NAc (Lowes et al., 2021; Fontaine et al., 2022). Aberrant levels of dopamine and serotonin are recognized as significant contributors to the pathogenesis of depression (Cabib and Puglisi-Allegra, 2012). Furthermore, emerging research is investigating the potential role of stress-induced neuroinflammation in depression, suggesting it could be a contributing factor (Calcia et al., 2016). Pro-inflammatory cytokines can interfere with neurotransmission and synaptic plasticity (Song et al., 1999; Nguyen et al., 2020; Zipp et al., 2023) and heightened neuroinflammation in the NAc has been documented in both depressive patients and animal models (Menard et al., 2017; Wang J et al., 2022). In essence, structural and functional changes, impaired synaptic plasticity, neurotransmitter dysregulation, and neuroinflammation within the NAc could result in an imbalance between reward and aversion. Elevated sensitivity to aversive stimuli, coupled with diminished responses to rewards, may underlie core symptoms of depression such as anhedonia, increased negative affect, and heightened stress responses (Nestler and Carlezon, 2006; Treadway and Zald, 2011; Warner-Schmidt et al., 2012; Felger and Treadway, 2017).

Interestingly, not all animals subjected to environmental stressors develop depression-like behaviors (Wood et al., 2010; Nasca et al., 2015). This observation has propelled research into the mechanisms that dictate diverse behavioral profiles in ‘resilient’ versus ‘susceptible’ animals (Cathomas et al., 2019). Investigations have delved into the transcriptional and epigenetic modifications that may contribute to stress susceptibility (Chandra et al., 2017; Labonte et al., 2017; Wang Z. J. et al., 2022; Kim et al., 2024). For example, Shisa6, an AMPA receptor auxiliary protein, is more abundant in the D1-MSNs of susceptible mice compared to the resilient ones (Kim et al., 2021). Enzymes like Dot1l and Kdm2b, which selectively regulate the demethylation of histone H3 lysine 79 (H3K79me2) in D2-MSNs, have been implicated in early-life stress-induced susceptibility (Kronman et al., 2021). Studies interested in the circuitry mechanism of stress susceptibility have primarily concentrated on the neural pathways involving dopaminergic neurons (Koo et al., 2019; Zhang et al., 2019). It has been suggested that enhanced phasic firing of the VTA ➔ NAc pathway, as opposed to the VTA ➔ mPFC pathway, mediates vulnerability to chronic social defeat stress (CSDS) (Chaudhury et al., 2013; Friedman et al., 2014). It is worth pointing out that both behavioral and physiological differences might present as traits before stress exposure. Altered performance in novel environment exploration and passive avoidance tests has been reported to predict outcomes after chronic social defeats (Milic et al., 2021). In addition, mice that later become resilient might show increased baseline activity in D1-MSNs and enhanced calcium responses to social interaction (Muir et al., 2018), which is in accordance with the finding that enhancing activity in D1-MSNs results in resilient behaviors (Francis et al., 2015). Although the evidence is preliminary, it shows the potential for early preventive interventions.

Antidepressants, at least partially, work by normalizing NAc functionality to improve mood and motivation (Tan et al., 2020; Carboni and Carta, 2022). Agents like ketamine, known for their rapid action, have demonstrated efficacy in restoring synaptic plasticity and normalizing structural outgrowth in the NAc (Belujon and Grace, 2014; Abdallah et al., 2017; Nardou et al., 2023). Furthermore, deep brain stimulation targeting the NAc has shown promise as a treatment for depression (Bewernick et al., 2010; Scangos et al., 2021; Song et al., 2024). A recent study demonstrated that augmenting the rewarding effects of relief from stress by stimulating DA inputs to the NAc or providing animals with food rewards promotes resilience (Dong et al., 2023). Thus, a deeper understanding of reward processing in the NAc could pave the way for novel therapeutic strategies to treat depression (Nestler and Carlezon, 2006).

## 8 Discussion

Recent research underscores the complex and dynamic role of the NAc in mediating both reward and aversion. This review has scrutinized the heterogeneous patterns of neuronal activity within the NAc during motivated behaviors. A particular focus has been the innovative use of fiber photometry and genetically encoded fluorescent sensors in the NAc. These technologies have shed light on the roles of neurotransmitters such as dopamine, serotonin, and acetylcholine in orchestrating the delicate balance of reward and aversion. Such methods have enabled the real-time observation of neurotransmitter dynamics in active animals, which paves the way for detailed dissections of the signaling pathways that regulate NAc function and its dysfunctions with an unprecedented level of precision. While this review has not delved into the function of NAc glia cells, it is worth acknowledging the increasing interest in their roles in the regulation of emotion and behavior (Adeluyi et al., 2019; Corkrum et al., 2020; Reverte et al., 2024). Future research should not only probe deeper into neuronal activity but also the NAc’s microenvironment, which encompasses factors such as extracellular matrix changes, neurovascular interactions, and the impact of peripheral signals on central reward mechanisms (Menard et al., 2017; Zhang et al., 2020; Hazlett et al., 2024).

Contemporary advancements in human neuroimaging techniques, such as functional magnetic resonance imaging (fMRI) and positron emission tomography (PET), continue to emphasize the role of the NAc in addiction and depression (Huang et al., 2024). The burgeoning field of optogenetics and chemogenetics in animal research presents promising avenues for teasing apart the complex networks involved in psychiatric disorders. Bridging the gap between animal research and human clinical studies through translational research is crucial in validating findings and ensuring their relevance to clinical populations. This review has synthesized the present knowledge of the involvement of the NAc in addiction and depression, mainly drawing from rodent studies. The neuropathological features of these conditions should not be viewed as mere opposites, but as conditions that may involve common molecular and circuitry pathways (Zhu et al., 2023a). The comorbidity of these two diseases is often overlooked in research. For instance, individuals with substance abuse frequently experience depressive episodes during withdrawal that can trigger drug-seeking and relapse. The precise role that the NAc plays in the progression of these complex interactions remains to be fully elucidated. Additionally, there is emerging evidence for the use of psychostimulants as a treatment for depression (Willyard, 2022; Calder and Hasler, 2023), however, discerning their antidepressant benefits from their addictive risks is still not well-defined. The potential mechanisms of stress susceptibility in depression have been discussed in the review, but a research gap should be noted in understanding the mechanisms that may predispose individuals to addiction (Volkow et al., 2019).

The critical role of the NAc in processing aversion underscores its broader significance in psychiatric disorders. Functional imaging studies have revealed that patients with posttraumatic stress disorder (PTSD) exhibit reduced responses to rewards and increased activity in the left accumbens when exposed to trauma reminders (Liberzon et al., 1999; Sailer et al., 2008; Pessin et al., 2021; Ploski and Vaidya, 2021). NAc circuits are implicated in both the learning and extinction of fear (Correia et al., 2016; Smith et al., 2021). In anxiety disorders, dysfunction of the NAc is associated with heightened fear and stress responses (Kalin et al., 2005; Ray et al., 2020; Xiao et al., 2021). Additionally, the NAc plays a significant role in pain processing, with its activity correlating with variations in pain intensity and unpleasantness (Kaneko et al., 2017). The NAc integrates multiple neurotransmitter systems, including glutamate, GABA, dopamine, opioids, and substance P, each contributing to pain and fear modulation (Harris and Peng, 2020; Belilos et al., 2023). Beyond aversion perception dysregulation, the NAc is involved in the development of maladaptive avoidance behaviors, highlighting its integral role in the emotional and motivational aspects of mental health.

In conclusion, there is a pressing need for further research to unravel the nuances of reward and aversion processing in the NAc. Such understanding is critical for the development of targeted therapeutic approaches for psychiatric disorders.

## Author contributions

YX: Writing – original draft, Writing – review & editing. YL: Writing – original draft, Writing – review & editing. MY: Funding acquisition, Writing – review & editing. KZ: Conceptualization, Funding acquisition, Writing – original draft, Writing – review & editing.
